# Parboiled Paddy Drying with Different Dryers: Thermodynamic and Quality Properties, Mathematical Modeling Using ANNs Assessment

**DOI:** 10.3390/foods9010086

**Published:** 2020-01-13

**Authors:** Ebrahim Taghinezhad, Antoni Szumny, Mohammad Kaveh, Vali Rasooli Sharabiani, Anil Kumar, Naoto Shimizu

**Affiliations:** 1 Department of Agricultural Technology Engineering, Moghan College of Agriculture and Natural Resources, University of Mohaghegh Ardabili, Ardabil 56199-11367, Iran; 2 Department of Chemistry, Wroclaw University of Environmental and Life Science, CK Norwida 25, 50-375 Wrocław, Poland; 3 Faculty of Agriculture and Natural Resources, University of Mohaghegh Ardabili, Ardabil 56199-11367, Iran; sirwankaweh@uma.ac.ir (M.K.); vrasooli@uma.ac.ir (V.R.S.); 4 Department of Mechanical Engineering, Delhi Technological University, Delhi 110042, India; anilkumar76@dtu.ac.in; 5 Research Faculty of Agriculture, Hokkaido University, Hokkaido 064-8589, Sapporo, Japan; shimizu@bpe.agr.hokudai.ac.jp

**Keywords:** parboiled paddy, thermodynamic, quality, Artificial Neural Network, mathematical modeling

## Abstract

The effect of hybrid infrared-convective (IRC), microwave (MIC) and infrared-convective-microwave (IRCM) drying methods on thermodynamic (drying kinetics, effective moisture diffusivity coefficient (*D_eff_*), specific energy consumption (SEC)) and quality (head rice yield (HRY), color value and lightness) characteristics of parboiled rice samples were investigated in this study. Experimental data were fitted into empirical drying models to explain moisture ratio (MR) variations during drying. The Artificial Neural Network (ANN) method was applied to predict MR. The IRCM method provided shorter drying time (reduce percentage = 71%) than IRC (41%) and microwave (69%) methods. The *D_eff_* of MIC drying (6.85 × 10^−11^–4.32 × 10^−10^ m^2^/s) was found to be more than the observed in IRC (1.32 × 10^−10^–1.87 × 10^−10^ m^2^/s) and IRCM methods (1.58 × 10^−11^–2.31 × 10^−11^ m^2^/s). SEC decreased during drying. Microwave drying had the lowest SEC (0.457 MJ/kg) compared to other drying methods (with mean 28 MJ/kg). Aghbashlo’s model was found to be the best for MR prediction. According to the ANN results, the highest determination coefficient (*R*^2^) values for MR prediction in IRC, IRCM and MIC drying methods were 0.9993, 0.9995 and 0.9990, respectively. The HRY (from 60.2 to 74.07%) and the color value (from 18.08 to 19.63) increased with the drying process severity, thereby decreasing the lightness (from 57.74 to 62.17). The results of this research can be recommended for the selection of the best dryer for parboiled paddy. Best drying conditions in the study is related to the lowest dryer SEC and sample color value and the highest HRY and sample lightness.

## 1. Introduction

Rice (*Oryza sativa* L.) is the major food for over half of the world’s population. Fajr is the most consumed and exported rice variety in Iran, although it has a low milling efficiency. The parboiling process has been used to resolve this problem. The parboiling process is the hydrothermal treatment of paddies before milling and involves three basic stages: soaking, steaming, and drying [1].

Several drying methods—superheated-steam, vacuum, hot-air, sun, and fluidized bed drying—have been applied for drying parboiled rice [2]. Hot air (HA) drying is an easy and conventional method for drying food materials. However, it has the limitations of longer drying time and low energy conversion efficiency [3]. Infrared (IR) and microwave (MIC) drying methods ensure rapid and efficient distribution of heat throughout the material. The advantages of these methods are energy efficiency, and short drying time [4].

The hybrid infrared-convective drying produces a synergistic effect, and is therefore considered to be more efficient than individual IR or convection drying methods [5]. It has been extensively applied on different products, including sweet potato [6] and kiwifruit [7]. Hybrid MIC-convection drying has been applied on different products such as lemon [8] and apple [9]. This technique leverages the advantages of both convection and MIC drying methods. Hybrid drying is a new technique in food drying.

Intermittent drying is a periodic drying technique and has many advantages when compared to continuous drying. This is because the moisture gradient and drying rate decreases and increases respectively during tempering [10]. Recently, intermittent drying has been widely applied in the paddy drying industry. Intermittent drying has been recommended for paddy drying by different researchers [11,12].

Mathematical modeling provides an instrumentation that enables drying rate and efficiency to be predicted under a range of conditions. Precise prediction can result in the best quality of the final product, as well as the reduction of process time [13]. Mathematical models proposed to explain the drying behavior of agricultural products commonly fall into three categories: theoretical, semi-empirical, and empirical models [14].

Artificial Neural Networks (ANN) have recently received much attention for the simulation of drying processes. ANN is used for adequate and precise control of the drying process. This method has been extensively used by many researchers [15,16]. The three main constituents of a typical ANN system are the learning rule, the transfer function, and the network architecture [17]. The two main types of ANN are cascade and feed-forward [13]. The advantage of ANN over mathematical modeling is its ability to process large amounts of noise from nonlinear and dynamic systems, especially when the underlying physical relationships are not fully understood [18]. ANNs include the neurons or nodes. In a multilayer network, the inputs from other related units are received using each neuron by means of a function of activation. As a result, it produces an output [19]. Some researchers have recently focused on the ANN and mathematical models in drying kinetics of agriculture products, such as eggplant [16], quince slices [20], sour cherry [21], Mentha spicata L. [22].

As per the literature review, very limited work has been performed on thermodynamic and quality properties, and mathematical and ANN modeling of parboiled paddy drying in hybrid infrared-convective (IRC), microwave (MIC) and infrared-convective-microwave (IRCM) dryers. The aim of this study was to analyze the effect of various techniques of drying on thermodynamic (drying kinetics, *D_eff_*, SEC) and quality (HRY, color value and lightness) properties of parboiled samples. Drying kinetic modeling and application ANN modeling are also done for prediction of moisture ratio in IRC, MIC, and IRCM drying methods. The best drying conditions for parboiled paddy were selected from the study results, which can be used to design energy efficient parboiled paddy drying system.

## 2. Materials and Methods

### 2.1. Sample Preparation

Paddy samples (var. Fajr) were purchased from Research Center of Rice in Mazandaran province of Iran. In general, amylose value and moisture content of samples were about 22.9% and 11 ± 1% (w.b.), respectively. The initial moisture content (MC) and dried weight of paddy samples was determined by the use of oven-drying technique (130 °C for 24 h in three replications) [23].

**Figure 1:**
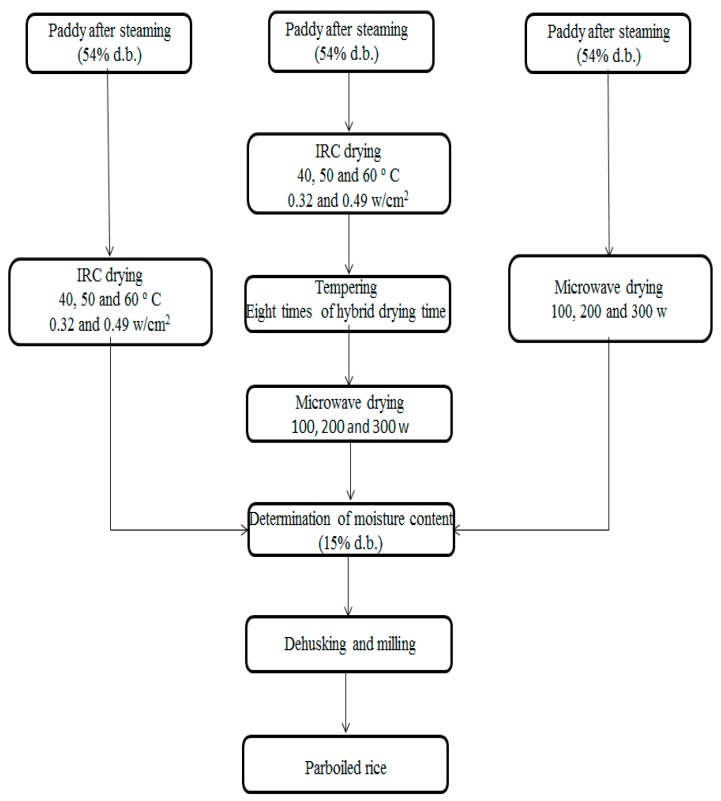
Experimental design for parboiled paddy drying (d.b.: dry basis).

The parboiling process involves three stages: soaking, steaming and drying. Figure 1 shows the complete experimental design for parboiled paddy drying.

(A) Soaking: Samples were soaked for 180 min in a bath with well-stirred water at a temperature of 65 ± 0.5 °C. Parboiled paddy (var. Fajr) has best quality at a temperature of 65 °C with a time of 4 min for the parboiled rice [1].

(B) Steaming: Following soaking, the paddy samples (1 kg) were drained and cooled down for 2 h at ambient temperature. The samples were steamed for 10 min and placed on a pot of a metal mesh at 96 °C temperature with 10 L of water boiling [24].

(C) Drying: After steaming, the paddy samples were dried using experimental drying different methods (IRC drying, IRCM drying, and MIC drying) for MC reduction in samples to 15% (d.b.). MC of samples is computable during drying based on sample dried weight using common equations.

### 2.2. Drying Techniques

An experimental setup of an IRCM dryer was manufactured and installed for parboiled paddy drying at the Department of Biosystems Engineering, Mohaghegh Ardabili University, Ardabil, Iran (Supplementary Figure S1). Temperature, velocity, humidity and sample weight were measured by means of a thermometer (Lutron Company, Taipei, Taiwan), anemometer (Lutron-YK Company, Taipei, Taiwan), hygrometer (Testo Company, Lenzkirch, German), and digital balance (A&D, Tokyo, Japan, ±0.01 g), respectively. Each drying experiment was conducted in three replications. The mean relative humidity and temperature of ambient were 24 ± 3% and 23 ± 2 °C, respectively.

### 2.3. Hybrid Infrared-Convection (IRC)

The paddy sample was transferred to a hybrid IRC dryer after steaming. The experiment was performed at different radiation intensity values (0.32 and 0.49 W/cm^2^), hot air temperatures (40, 50 and 60 °C), and a constant airflow rate (1 m/s). To determine the infrared power, the distance between the emitter and the sample was changed. The distances between the infrared lamp and samples were 20 and 10 cm for 0.32 and 0.49 W/cm^2^, respectively. The samples were spread on a 10 × 10 cm surface.

### 2.4. Infrared Convective-Microwave (IRCM)

In general, tempering duration was eight times longer than the drying time at the ambient temperature [25]. The drying process was performed in two stages: (I) IRC drying was conducted at the first stage (at 40, 50 and 60 °C using 0.32 and 0.49 W/cm^2^ and 1 m/s velocity) to reduce MC from 54 to 27% (d.b.); and (II) the second stage included MIC drying (100, 200 and 300 W) to drop sample MC from 27 to 15% (d.b.).

### 2.5. Microwave (MIC)

A programmable microwave oven (M945, Samsung Electronics Inc., Ridgefield Park, NJ, USA) with a 1000 W maximum output at 2450 MHz was applied for the drying experiment. The dimensions of the oven were 330 × 370 × 210 mm^3^. Three power levels (100, 200 and 300 W) were used to study MC reduction in samples from 54 to 15% (d.b.).

### 2.6. Drying Kinetics Modeling

MC values of paddy were calculated and obtained at several equal time intervals during drying:

(1) $$MC=\frac{\left(\left(W_{t}-W\right)-W_{e}\right)}{W_{e}}$$

where *MC* is the moisture content (g_water_/g_dry matter_), *W* is the amount of evaporated moisture (g), *W_t_* is the initial weight of sample (g), *W_e_* is the dry matter content of sample (g).

The moisture ratio (*MR*) for parboiled paddy was found using Equation (2) [26]:

(2) $$MR=\frac{\left(M_{t}-M_{e}\right)}{\left(M_{i}-M_{e}\right)}$$

where *MR* is the moisture ratio (decimal), *M_t_* is the moisture content (% d.b.), *M_e_* is the equilibrium moisture content (% d.b.), *M_i_* is the initial moisture content (% d.b.). The equilibrium *MC* of parboiled paddy (long-grain) was calculated using the method of Ondier et al. They suggested the modified equation of Chung-Pfost as [27]:

(3) $$M_{e}=\frac{-1}{C}\ln\left[\frac{-\left(T_{c}+B\right)\times \ln\left(RH\right)}{A}\right]$$

where *RH* is the relative humidity (%), *T_c_* is the hot air temperature (°C), and *A*, *B*, and *C* for parboiled paddy are 406.92, 23.6172 and 0.2303, respectively. The equilibrium moisture content (*M_e_*) was found to be 7.9% (d.b.).

### 2.7. Mathematical Modeling

Drying curves of parboiled paddy samples were obtained for three different drying processes (IRC, IRCM and MIC drying) and fitted into five different models (Table 1). Where the coefficients a, b, k, and n are empirical constants and coefficients in drying models and t and MR are drying time and moisture ratio, respectively. The curve fitting tool “Curve Expert (Ver. 1.4)” and the technique of nonlinear regression were used to fit the best models into the MR data. This model, which is MR versus drying time (*t*), is called kinetic drying. The statistical factors, which include the coefficient of determination (*R*^2^), chi-square (χ^2^) and root mean square error (*RMSE*), were used for evaluation and comparison of models. The model with the highest *R*^2^ and the lowest χ^2^ and *RMSE* was chosen as the best model for estimating the drying curves [28,29].

### 2.8. Determination of Effective Moisture Diffusivity (D_eff_)

The effective diffusivity of (liquid) moisture transport is a fundamental parameter. Drying rates are represented using empirical models of explicit time-dependent functions. It is a simple and accurate analysis which can be performed using one-dimensional diffusion and Fick’s diffusion equation. Fick’s second law of diffusion with spherical coordinates was used to calculate the *D_eff_* and thermal diffusion. The effective moisture diffusivity determined in the thickness steady-state and transient conditions. The analytical solution for single-layer objects and several geometries was explained by Amiri Chayjan (2011), according to Equation (4) [31]:

(4) $$MR=\frac{M_{t}-M_{e}}{M_{i}-M_{e}}=\frac{6}{\pi^{2}}\overset{\infty }{\underset{n=1}{\sum }}\frac{1}{n^{2}}\exp\left(-\frac{n^{2}\pi^{2}}{r^{2}}D_{eff}t\right)$$

where *D_eff_* is the effective moisture diffusivity (m^2^/s), *t* is the drying time (s), *r* is the radius of paddy (m), *n* is the number of training patterns.

Equation (4) can be simplified to the first term of the series for a long drying time:

(5) $$MR=\left(\frac{6}{\pi^{2}}\right)\exp\left(-\frac{\pi^{2}D_{eff}t}{r^{2}}\right)$$

Thew *D_eff_* as also obtained typically by means of the slope of Equation (5). The slope (*k*) was computed using a plotting of ln(*MR*) versus time.

(6) $$k=\left(\frac{D_{eff}\pi^{2}}{r^{2}}\right)$$

### 2.9. Specific Energy Consumption

#### 2.9.1. IRC Drying

This dryer uses different sources of energy including thermal energy (infrared lamps, heaters) and mechanical energy (blowers). Thermal energy was determined using Equation (7) [31]:

(7) $$EU_{ther}=\left(K+\rho_{a}\cdot v\cdot A\cdot C_{a}\cdot \Delta T\right)\cdot 3600\cdot t$$

where *EU_ther_* is the thermal energy consumption (kJ), *K* is the lamp power (W), *ρ_a_* is the density of air (kg/m^3^), *v* is the air velocity (m/s), *A* is the cross sectional area of container in which sample was placed (m^2^), *C_a_* is the specific heat of air (kJ/kg °K), Δ*T* is the temperature difference (°C).

The mechanical energy consumed by the blower was obtained by Equation (8) [32]:

(8) $$EU_{mec}=\Delta P\cdot M_{air}\cdot t$$

where *EU_mec_* is the mechanical energy consumption (kJ), *P* is the pressure difference (mbar), *M_air_* is the drying air flow rate (m^3^/s).

#### 2.9.2. MIC Drying

The SEC value of the MIC dryer was computed using Equation (9) [33].

(9) $$EU_{ther}=P_{mic}\cdot t\cdot 3600$$

where *P_mic_* is the microwave power (kW).

#### 2.9.3. IRCM Drying

The total *SEC* values in the infrared-convection-microwave dryer was calculated from the sum of *SEC* of infrared, convection and microwave dryer (thermal energy and mechanical energy) [8]:

(10) $$EU_{mec}=\left(A\cdot v\cdot \rho_{a}C_{a}\cdot \Delta T+K\cdot t+P_{mic}\cdot t\right)\cdot 3600$$

The *SEC* for drying of 1 kg of paddy for these three dryers was calculated using Equation (11) [6]:

(11) $$SEC=\frac{EU_{ther}+EU_{mec}}{M_{W}}$$

where *SEC* is the specific energy consumption (kJ/kg), *M_W_* is the weight of lost water (kg).

### 2.10. Artificial Neural Networks (ANN) Modeling

Different multilayer feed-forward-back-propagation (FFBP) and cascade-forward-back-propagation (CFBP) networks were developed and tested, using various hidden layers (1 and 2) and neurons, to determine the best network. Several topologies were investigated using neuron changes in the hidden layer. Training in these networks is an iterative process. The training process is validated if the error between the predicted and desired values is minimum. The optimization method was applied in order to select layers and neurons to evaluate various topologies. An ANN was separately specified for each dryer.

#### 2.10.1. ANN 1: IRC DRYER

Three input parameters were used in the experiments with the IRC dryer. The MR values were measured. Networks with one neuron in the output layer (MR) and three neurons in the input layer (air temperature (°C), radiation intensity (W/cm^2^), and drying time (min)) were designed. In this stage, 163 data points were used for ANN. In the first group, 30% (48) was selected for testing, and in the second group, 70% of the data (115) was randomly selected from the entire data pool for training.

#### 2.10.2. ANN 2: IRCM Drying

IRCM drying had four input variables including air temperature (°C), radiation intensity (w/cm^2^), microwave power (W), and drying time (min). The output variable for evaluating drying performance was MR. Therefore, the input layer and output layer consisted of four and one neurons respectively. In this research, 70% (315) of the data was randomly applied for training and 30% (135) was used for testing of neural networks.

#### 2.10.3. ANN 3: MIC Drying

For MIC drying, networks with two neurons (microwave power (W) and drying time (min)) in the input layer and one neuron in the output layer (MR) were designed. About 70% (49) of the data were used for training to obtain the best topology.

The ANNs were also trained with Levenberg-Marquardt (LM) and Bayesian Regulation (BR) learning algorithms. Three transfer operations (Pureline, Tansig and Logsig) were applied to reach the suitable topology [29].

(12) $$Y_{j}=X_{j}\;(\mathrm{Pureline})$$

(13) $$Y_{j}=\frac{2}{\left(1+\exp\left(2X_{j}\right)\right)-1}\;(\mathrm{Tansig})$$

(14) $$Y_{j}=\frac{1}{1+\exp\left(-X_{j}\right)}\;(\mathrm{Logsig})$$

where *X_j_* are the weighted inputs for each neuron in the *j*th layer and are calculated as follows:

(15) $$X_{j}=\overset{m}{\underset{i=1}{\sum }}W_{ij}\times Y_{i}\times b_{j}$$

where *W_ij_* is the weight between *i*-th and *j*-th layers, *X_j_* is the *j*-th input neuron, *Y_i_* is the *j*-th output neuron, *b_j_* is the bias of *j*-th neuron for FFBP and CFBP.

The mean squared error (*MSE*) was computed using Equation (18) [34]:

(16) $$MSE=\frac{1}{N_{0}}\overset{N}{\underset{i=1}{\sum }}{\left(S_{k}-T_{k}\right)}^{2}$$

where *S_k_* is the network output for *k*th pattern, *T_k_* is the target output for *k*th pattern, *N*_0_ is the number of output neurons.

The efficiency of the ANN models were quantified using the *R*^2^ and the *MAE* between the real and predicted values that were calculated according to the following equations [28]:

(17) $$R^{2}=1-\frac{\sum_{k=1}^{N}\left[S_{k}-T_{k}\right]}{\sum_{k=1}^{N}\left[S_{k}-\frac{\sum_{k=1}^{n}S_{k}}{n}\right]}$$

(18) $$MAE=\frac{100}{N}\overset{n}{\underset{k=1}{\sum }}\left|\frac{S_{k}-T_{k}}{T_{k}}\right|$$

*MAE*—mean absolute error. 

### 2.11. Quality Properties

#### 2.11.1. Head Rice Yield (HRY) 

After four days of drying, paddy was de-husked using laboratory stake rubber roller rice husker instrument (Satake Company, Hiroshima, Japan). Then the polishing of samples was performed by a polisher (Satake Company, Hiroshima, Japan) for 90 S. The HRY was computed with dividing of whole grains weight to paddy; the average of triplicates was considered [35].

#### 2.11.2. Color Value and Lightness

To measure the color and lightness, precision color reader (Model 4510, Reston Company, Fairfax, VA, USA) was used. Before measurement, the instrument was calibrated with white and black plates. The results were expressed in term of L*, a*, b* values. L* shows brightness from black to white, positive and negative “a*” values represent redness/greenness, and “b*” value represents yellowness/blueness. The color measurement were done in ten replication and the average values were subjected to further analysis. The parboiled rice color value (B) was calculated using Equation (19) [36].

(19) $$\mathrm{Colorvalue}=\sqrt{a^{*2}+b^{*2}}$$

## 3. Results and Discussion

### 3.1. Drying Time

The values of moisture ratio versus drying time for different drying conditions are shown in Supplementary Figure S2. As can be seen, MR decreases continuously and exponentially with increasing drying time.

#### 3.1.1. Hybrid IRC Drying 

Moisture removal from samples was accelerated when air temperature and radiation intensity increased. As a result, the drying time decreased from 68 to 40 min.

#### 3.1.2. IRCM Drying

The drying time of parboiled paddy samples using 0.32 and 0.49 W/cm^2^, 40–60 °C and 100–300 W conditions varied between 24 and 82 min. The drying time reduced with increase in the radiation intensity, air temperature, and MIC power.

#### 3.1.3. Microwave Drying

The results showed that drying time decreased (72–22 min) when MIC power increased. This result is in agreement with the results of other studies [29]. In addition, the increased radiation intensity or air temperature or microwave power also increased the product surface temperature and moisture loss, leading to faster drying [37].

### 3.2. Mathematical Modeling

Five various empirical models were applied to analyze the MR data of parboiled paddy in various dryers and results are presented in Supplementary Table S1. For all dryers, Aghbashlo’s model had the highest *R*^2^ (0.99) and the lowest *RMSE* (0.0194) and χ^2^ (0.0004) values. Therefore, Aghbashlo’s model was chosen as the best model for explaining the drying properties of parboiled paddy in IRMC dryer. These results were in agreement with other researcher’s reported data [32,38].

### 3.3. Effective Moisture Diffusivity (Deff)

**Figure 2:**
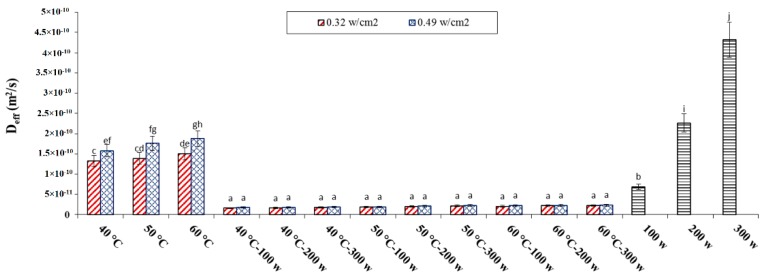
The values of *D_eff_* versus air temperature (°C), different infrared radiations (W) and microwave power (W) for rice drying under different dryers. Note: The same letter over column shows that the mean amount had no significant difference (*p* < 0.05) based on Duncan’s test. The error bars represent standard error of the means.

*D_eff_* values of the parboiled paddy samples dried by different drying techniques are shown in Figure 2. In hybrid IRC drying, the lowest (1.32 × 10^−10^ m^2^/s) and highest (1.87 × 10^−10^ m^2^/s) *D_eff_* values belonged to the samples dried at 40 °C using 0.32 W/cm^2^ radiation and at 60 °C using 0.49 W/cm^2^ radiation, respectively. In IRCM drying, the lowest (1.58 × 10^−11^ m^2^/s) and highest (2.31 × 10^−11^ m^2^/s) *D_eff_* values belonged to the samples dried at 40 °C using 0.32 W/cm^2^ and 100 W and at 60 °C using 0.49 W/cm^2^ and 300 W, respectively. At the same time, in MIC drying, the lowest (6.85 × 10^−11^ m^2^/s) and highest (4.32 × 10^−10^ m^2^/s) *D_eff_* were reported for samples dried at 100 and 300 W power levels, respectively. For all dryers, the *D_eff_* values increased with increase in the drying temperature, radiation intensity, and MIC power. High temperatures increased the molecular activity of water and caused higher diffusivity. According to Figure 2, *D_eff_* in IRCM drying was lower than in hybrid and microwave dryers. Aydogdu et al. [39] proved that effective moisture diffusivity for eggplant in HA drying (5.07 × 10^−10^ m^2^/s) is lower than that observed in microwave-infrared drying (7.10 × 10^−9^–1.44 × 10^−8^ m^2^/s) [39]. However, the highest *D_eff_* was observed in samples dried using microwave drying compared to other drying techniques, because the high MIC power can accelerate the evaporation of water molecules in paddy samples and provide a faster decrease in the rice MC corresponding to the higher value of D_eff_ [40]. *D_eff_* results were comparable with the values reported by other authors [41]. The reported *D_eff_* values were within the general range of 10^−8^ to 10^−12^ m^2^/s for agricultural and food products [29].

### 3.4. Specific Energy Consumption (SEC)

**Figure 3:**
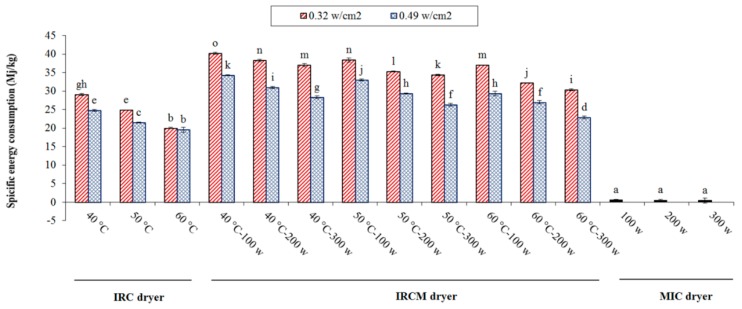
Specific energy consumption for rice drying under different dryers. Note: The same letter over column shows that the mean amount had no significant difference (*p* < 0.05) based on Duncan’s test. The error bars represent standard error of the means.

The values of SEC for different dryers are shown in Figure 3. It was observed that SEC values decreased as radiation intensity, drying temperature or microwave power increased. This is because low radiation intensity, air temperature, and microwave power can cause a relative decrease in *D_eff_*, leading to an increase in SEC values. For IRC drying, the highest (29.06 MJ/kg) and lowest (19.55 MJ/kg) SEC values were obtained at 40 °C using 0.32 W/cm^2^ and at 60 °C using 0.49 W/cm^2^, respectively. In IRCM drying, the highest (40.23 MJ/kg) and lowest (22.88 MJ/kg) SEC values were observed at 40 °C using 0.32 W/cm^2^ and 100 W and at 60 °C using 0.49 W/cm^2^ and 300 W, respectively. According to Figure 3, the lowest SEC in microwave drying was 0.457 kWh/kg, which occurred at 100 W of microwave power, whereas the SEC value of 0.575 MJ/kg was observed when using the microwave power of 300 W. In other words, the highest SEC was 1.25 times greater than the lowest energy required. The reduction of SEC was related to the effect of volumetric heating of MIC, which substantially decreased the duration of drying. These SEC values are comparable with the values reported by other researchers [42,43]. The SEC in the IRCM dryer was higher than that in the hybrid IRC and MIC dryers. Similar results were reported by other researchers [44].

### 3.5. ANN Modeling

Table 2 shows the best ANN topologies, threshold functions, and different algorithms applied for predicting MR in the drying of parboiled paddy samples using different dryers. The highest *R*^2^ between ANN-predicted MR values of paddy samples in different dryers and their measured values for training and testing datasets was >0.99.

#### 3.5.1. IRC Drying

As shown in Table 2, the neural network with the 3-8-8-1 topology, TAN, PUR, and TAN transfer functions, and the LM training algorithm (trainlm) had the lowest MAE values (0.0059 for training and 0.0048 for testing) and the highest *R*^2^ (0.9992 for training and 0.9993 for testing).

#### 3.5.2. IRCM Drying

The best model for prediction of MR belonged to the cascade-forward-back-propagation (CFBP) network, the 4-10-10-1 topology and the TANSIG threshold function with the BR algorithm in the first strategy.

#### 3.5.3. Microwave Drying

The widely used topologies and threshold functions have a good training process. The feed-forward back-propagation (FFBP) structure with TAN-TAN-TAN threshold function and the BR algorithm with 2-10-10-1 topology had the lowest MSE (0.00065), MAE (0.0054 for test and 0.0073 for training) values and the highest *R*^2^ (0.9990 for testing and 0.9989 for training). These findings are in agreement with those reported by another author [7].

### 3.6. Quality Properties

#### 3.6.1. Head Rice Yield (HRY)

**Figure 4:**
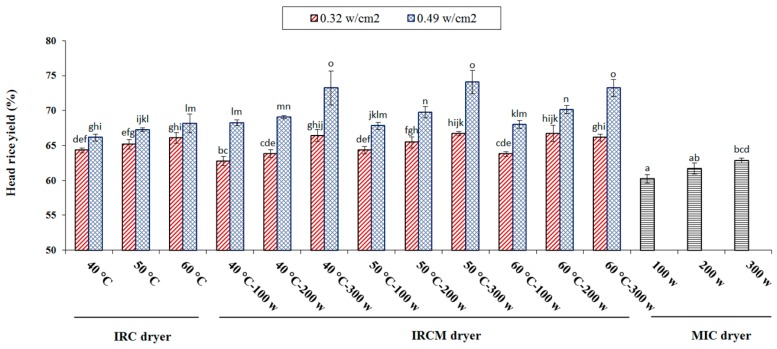
The HRY affected by different drying methods for parboiled rice drying. Note: The same letter over column shows that the mean amount had no significant difference (*p* < 0.05) based on Duncan’s test. The error bars represent standard error of the means.

As shown in Figure 4, HRY values were between 60.2 and 74.07%. The maximum HRY (74.08%) was obtained when rice was dried using IRCM drying method (at 50 °C and 300 W). Taghinezhad and Brenner [1] found the maximum HRY (67.37%) after steaming to be 65 °C with a soaking time of 6 min [1]. The minimum HRY was observed (60.21%), when paddy was dried using MIC drying method (100 W). According to Figure 4, increasing drying temperature (from 40 °C to 60 °C) and microwave power (from 100 to 300 W) caused an increase of *HRY*. The amount of *HRY* is likely to be related to the higher temperatures [45]. Similar reports related to the effect of the drying conditions on the *HRY* have also been presented by other researchers [46]. They reported that the *HRY* of long-grain SP 1 parboiled rice was relatively higher when drying temperature increased. The experimental results for HRY agreed with results reported by other researchers [47]. They reported the HRY values in the range 60–80% for parboiled rice (KDML 105 paddy). Different drying systems have significant influence on the extent of HRY for parboiled rice as shown in Figure 4. Therefore, it seems to be established that an increase in drying temperature and power at the range we investigated in the present research may lead to an increase in the degree of starch gelatinization (DSG). This is because high temperature and radiation penetrates the rice grain kernel and leads to greater DSG [46]. Hence the appropriate condition of drying can lead to increase in HRY [36]. Also, the increase in HRY could also be related to facilitating the separation of the gelatinized kernels from the husk following the drying. As a result, the milling becomes easier following this separation of the husk from the kernel [48].

#### 3.6.2. Color Value

**Figure 5:**
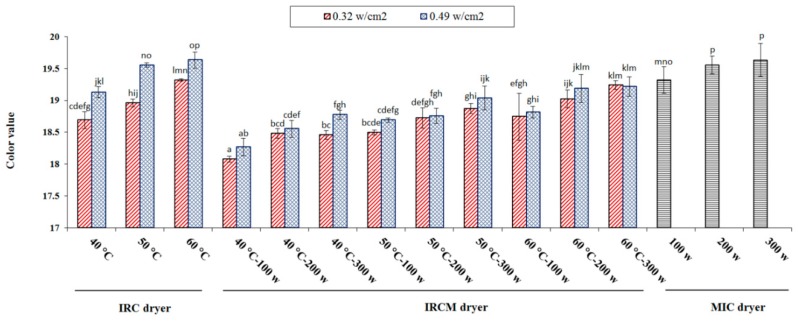
The color value affected by different drying methods for parboiled rice drying. Note: The same letter over column shows that the mean amount had no significant difference (*p* < 0.05) based on Duncan’s test. The error bars represent standard error of the means.

The color of rice is a very important indicator for measuring the physical properties of processed rice. Figure 5 signifies that color value is greatly affected by different drying methods. The lightness value ranges from 18.08 to 19.63. The darkest color value was observed for IRC dryer (60 °C). The discoloration of rice was caused by the reaction of released sugar with amino acid of grain treated under high temperature [49]. Similar results have been presented by other researchers [43] studying the effect of the different parboiling conditions on the color value. Also, many researchers evaluated the parboiled rice color [50,51], noting that final color changes during parboiling can be explained as non-enzymatic browning (Maillard reactions) and the drying variables influenced the intensity of color. In other words, the color parameters revealed that during parboiling, yellow and red bran pigments diffused from the bran into the endosperm. Bran pigments diffused into the endosperm affect the color of parboiled rice [52].

#### 3.6.3. Lightness

**Figure 6:**
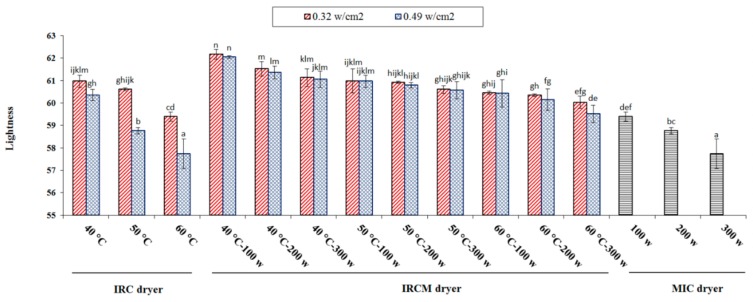
The lightness affected by different drying methods for parboiled rice drying. Note: The same letter over column shows that the mean amount had no significant difference (*p* < 0.05) based on Duncan’s test. The error bars represent standard error of the means.

The lightness value ranges from 57.74 to 62.17; these values were relatively similar to those reported by others [24]. The darkest rice was observed when rice was treated at 60 °C drying temperature (under IRC dryer). Figure 6 represents the effect of different drying methods on the lightness of parboiled rice. The degree of lightness decreased with the severity of drying process (increase of drying temperature (from 40 to 60 °C) and microwave power (from 100 to 300 W)). These findings are in agreement with those of other researchers [50,51]. Consequently, it can be explained that rice should not be treated under severe drying conditions. Higher temperature application could affect lightness of rice, leading to poor quality of rice and less demand in the market [53].

## 4. Conclusions

Drying is one of the commonly used methods for reducing product moisture, especially for long storage times. In this research, the effects of drying different methods on the thermodynamic and quality characteristics of parboiled paddy were evaluated in this study. Five mathematical methods were applied to predict the moisture ratio of parboiled rice. The results proved that with increasing of radiation intensity or air temperature or microwave power increased the product surface temperature and moisture loss. As a result, it led to faster drying. Aghbashlo’s model was the best model for the prediction of parboiled paddy MR. It was able to describe the thin-layer thermodynamic characteristics of samples in the three dryers. The highest *D_eff_* was obtained under the microwave drying conditions. The lowest SEC was calculated in MIC drying. ANN be able to predict the MR with high accuracy. The physical properties were influenced by means of different drying methods. The obtained results indicate that the amount of HRY and color value hardness increased during drying, while the amount of lightness decreased. The results of this research can be used for the selection of the best dryer in the parboiling industry of paddy. The best drying conditions were related to the highest HRY and sample lightness, and the lowest dryer SEC and sample color value.

## Figures and Tables

**Table 1:** Mathematical empirical models [30].

Models	Equation
Aghbashlo	$MR=\exp\left(\frac{-at}{1+bt}\right)$
Page	$MR=\exp\left(-kt^{n}\right)$
logistic	$MR=\frac{a}{1+b\;\exp\left(kt\right)}$
Demir	$MR=a\;\exp{\left(-kt\right)}^{n}+b$
Midili	$MR=a\;\exp(-kt^{n})+bt$

**Table 2:** The best selected topologies including training algorithm, different layers and neurons for FFBP and CFBP for moisture ratio in infrared convection, infrared-convective-microwave and microwave drying.

Drying Type	Network	Training Algorithm	Threshold Function	Number of Layers and Neurons	MSE	Train		Test	
						*R* ^2^	MAE	*R* ^2^	MAE
Infrared- convective drying	FFBP	LM	TAN-PUR-TAN	3-8-8-1	0.00057	0.9992	0.0059	0.9993	0.0048
	FFBP	BR	TAN-TAN-TAN	3-12-1	0.00145	0.9984	0.0109	0.9986	0.0089
	CFBP	LM	TAN-TAN-TAN	3-12-12-1	0.00101	0.9990	0.0080	0.9991	0.0064
	CFBP	BR	TAN-TAN-LOG	3-18-1	0.00062	0.9992	0.0067	0.9992	0.0052
Infrared- convective- microwave drying	FFBP	LM	TAN-TAN-LOG	4-15-15-1	0.00108	0.9982	0.0114	0.9983	0.0106
	FFBP	BR	LOG-TAN-PUR	4-5-5-1	0.00074	0.9987	0.0095	0.9988	0.0092
	CFBP	LM	TAN-TAN-TAN	4-8-1	0.00067	0.9991	0.0069	0.9991	0.0061
	CFBP	BR	TAN-TAN-TAN	4-10-10-1	0.00052	0.9994	0.0044	0.9995	0.0039
Microwave drying	FFBP	LM	PUR-TAN-TAN	2-20-1	0.00079	0.9986	0.0088	0.9988	0.0069
	FFBP	BR	TAN-TAN-TAN	2-10-10-1	0.00065	0.9989	0.0073	0.9990	0.0054
	CFBP	LM	TAN-TAN-TAN	2-10-10-1	0.00101	0.9981	0.0111	0.9982	0.0105
	CFBP	BR	TAN-LOG-PUR	2-15-10-1	0.00981	0.9985	0.0098	0.9987	0.0084

## Supplementary Materials

The following are available online at https://www.mdpi.com/2304-8158/9/1/86/s1, Figure S1: Schematic view of the experimental infrared convective-microwave drying: (1) fan and electric motor, (2) electrical heater, (3) duct and air tunnel, (4) infrared-convective chamber, (5) infrared lamp, (6) inverter and thermostat, (7) microwave chamber, (8) precision balance, (9) computer, (10) thermometer, (11) hygrometer, and (12) chassis, Figure S2: Effect of drying different methods on MR during drying of parboiled rice ((**a**): IRC drying; (**b**): IRCM drying; (**c**): MIC drying), Table S1: Statistical comparison for prediction of MR.

## Nomenclature

**Abbreviations**	
ANN	Artificial Neural Network
*b_j_*	The bias of *j*-th neuron for FFBP and CFBP
HA	Hot air
HRY	Head rice yield
IR	Infrared
IRC	Hybrid infrared-convective
IRCM	Infrared-convective-microwave
MIC	Microwave
MR	Moisture ratio
SEC	Specific energy consumption
w.b.	Wet bulb
*N*_0_	Number of output neurons
MAE	Mean absolute error
MSE	Mean squared error
RMSE	Root mean square error
**Symbols**	
A	Cross sectional area of container in which sample was placed (m^2^)
C	Specific heat (kJ/kg °K)
*D*_0_	Pre-exponential factor of the Arrhenius equation (m^2^/s)
*D_eff_*	Effective moisture diffusivity (m^2^/s)
EU	Energy consumption (kJ)
K	Lamp power
MC	Moisture content (g_water_ g^−1^_dry matter_)
MR	Moisture ratio (decimal)
M	Moisture content (% d.b.)
M_w_	Weight of loss water (kg)
*n*	number of training patterns
*N*	Number of observations
*N*_0_	Number of output neurons
P	Power (kW)
*R* ^2^	Determination coefficient
*r*	The radius of paddy (m)
RH	Relative humidity (%)
S_k_	Network output for *k*th pattern
t	Drying time (s)
T_c_	Air temperature (°C)
T_k_	Target output for *k*th pattern
*v*	Air velocity (m/s)
z	Number of drying constants
W	Amount of evaporated moisture (g)
W_t_	Initial weight of sample (g)
W_e_	Dry matter content of sample (g)
W_ij_	Weight of between *i*-th and *j*-th layers
X_i_	The *i*-th input neuron
Y_j_	The *j*-th output neuron
**Greek Symbols**	
χ^2^	Chi-square
*ρ*	Density (kg/m^3^)
Δ*T*	Temperature difference
Δ*P*	Pressure difference (mbar)
**Subscripts**	
a	air
ther	Thermal
mec	Mechanical
t	Total
i	Initial
e	Equilibrium
Exp,i	Experimental *i*th data
Pre,i	Predicted *i*th data

## References

[1] Taghinezhad E., Brenner T. (2017). Mathematical modeling of starch gelatinization and some quality properties of parboiled rice based on parboiling indicators using RSM. J. Food Process Eng..

[2] Swasdisevi T., Sriariyakula W., Tia W., Soponronnarit S. (2010). Effect of pre-steaming on production of partially-parboiled rice using hot-air fluidization technique. J. Food Eng..

[3] Del-Rosario A.J., VÍctor O.P., Abel C.G. (2019). Hot air drying kinetics of thin layers of prickly pear fruit paste. Sains Malays..

[4] Wu B., Ma H., Qu W., Wang B., Zhang X., Wang P., Wang J., Atungulu G.G., Pan Z. (2014). Catalytic infrared and hot air dehydration of carrot slices. J. Food Process Eng..

[5] Nejadi J., Nikbakht A.M. (2016). Numerical Simulation of Corn Drying in a Hybrid Fluidized Bed-Infrared Dryer. J. Food Process Eng..

[6] Onwude D.I., Hashim N., Abdan K., Janius R., Chen G. (2018). Investigating the influence of novel drying methods on sweet potato (*Ipomoea batatas* L.): Kinetics, energy consumption, color, and microstructure. J. Food Process Eng..

[7] Özdemir M.B., Aktaş M., Şevik S., Khanlari A. (2017). Modeling of a convective-infrared kiwifruit drying process. Int. J. Hydrogen Energy.

[8] Deepika S., Sutar P.P. (2018). Combining osmotic–steam blanching with infrared–microwave–hot air drying: Production of dried lemon (*Citrus limon* L.) slices and enzyme inactivation. Dry. Technol..

[9] Dehghannya J., Farshad P., Heshmati M.K. (2018). Three-stage hybrid osmotic–intermittent microwave–convective drying of apple at low temperature and short time. Dry. Technol..

[10] Kowalski S.J., Pawłowski A. (2010). Modeling of kinetics in stationary and intermittent drying. Dry. Technol..

[11] Assar M., Golmohammadi M., Rajabi-Hamaneh M., Hassankiadeh M.N. (2016). A Combined experimental and theoretical approach to study temperature and moisture dynamic characteristics of intermittent paddy rice drying. Chem. Eng. Commun..

[12] Ghasemi A., Sadeghi M., Mireei S.A. (2017). Multi-stage intermittent drying of rough rice in terms of tempering and stress cracking indices and moisture gradients interpretation. Dry. Technol..

[13] Kaveh M., Amiri Chayjan R., Nikbakht A.M. (2017). Mass transfer characteristics of eggplant slices during length of continuous band dryer. Heat Mass Transf..

[14] Doymaz I., Demir H., Yildirim A. (2015). Drying of quince slices: Effect of pretreatments on drying and rehydration characteristics. Chem. Eng. Commun..

[15] Prakash O., Kumar A. (2014). Application of artificial neural network for the prediction of jaggery mass during drying inside the natural convection greenhouse dryer. Int. J. Ambient. Energy.

[16] Prakash O., Kumar A., Kaviti A.K., Kumar P.V. (2015). Prediction of the rate of moisture evaporation from jaggery in greenhouse drying using the fuzzy logic. Heat Transf. Res..

[17] Jahed Armaghani D., Tonnizam Mohamad E., Hajihassani M., Yagiz S., Motaghedi H. (2016). Application of several non-linear prediction tools for estimating uniaxial compressive strength of granitic rocks and comparison of their performances. Eng. Comput..

[18] Aghbashlo M., Hosseinpour S., Mujumdar A.S. (2015). Application of artificial neural networks (anns) in drying technology: A comprehensive review. Dry. Technol..

[19] Saraceno A., Aversa M., Curcio S. (2012). Advanced modeling of food convective drying: A comparison between artificial neural networks and hybrid approaches. Food Bioprocess Technol..

[20] Chasiotis V.K., Tzempelikos D.A., Filios A.E., Moustris K.P. (2019). Artificial neural network modelling of moisture content evolution for convective drying of cylindrical quince slices. Comput. Electron. Agric..

[21] Chayjan R.A., Kaveh M., Khayati S. (2014). Modeling some drying characteristics of sour cherry (*Prunus cerasus* L.) under infrared radiation using mathematical models and artificial neural networks. Agric. Eng. Int. CIGR J..

[22] Karakaplan N., Goz E., Tosun E., Yuceer E. (2019). Kinetic and artificial neural network modeling techniques to predict the drying kinetics of *Mentha spicata* L.. J. Food Process. Preserv..

[23] AOAC (1995). Official Methods of Analysis.

[24] Islam M.R., Roy P., Shimizu N., Kimura T. (2002). Effect of processing conditions on physical properties of parboiled rice. Food Sci. Technol. Res..

[25] Aquerreta J., Iguaz A., Arroqui C., Virseda P. (2007). Effect of high temperature intermittent drying and tempering on rough rice quality. J. Food Eng..

[26] Seremet L., Botez E., Nistor O.-V., Andronoiu D.G., Mocanu G.-D. (2016). Effect of different drying methods on moisture ratio and rehydration of pumpkin slices. Food Chem..

[27] Ondier G.O., Siebenmorgen T.J., Bautista R.C., Mauromoustakos A. (2011). Equilibrium moisture contents of pureline, hybrid and parboiled rice. Am. Soc. Agric. Biol. Eng. (ASABE).

[28] Yang X.-H., Deng L.-Z., Mujumdar A.S., Xiao H.-W., Zhang Q., Kan Z. (2018). Evolution and modeling of colour changes of red pepper (*Capsicum annuum* L.) during hot air drying. J. Food Eng..

[29] Kaveh M., Amiri Chayjan R. (2017). Modeling thin-layer drying of turnip slices under semi-industrial continuous band dryer. J. Food Process. Preserv..

[30] Amiri Chayjan R., Amiri Parian J., Esna-Ashari M. (2011). Modeling of moisture diffusivity, activation energy and specific energy consumption of high moisture corn in a fixed and fluidized bed convective dryer. Span. J. Agric. Res..

[31] Aktas M., Sevik S., Aktekeli B. (2016). Development of heat pump and infrared-convective dryer and performance analysis for stale bread drying. Energy Convers. Manag..

[32] Torki-Harchegani M., Ghanbarian D., Ghasemi Pirbalouti A., Sadeghi M. (2016). Dehydration behaviour, mathematical modelling, energy efficiency and essential oil yield of peppermint leaves undergoing microwave and hot air treatments. Renew. Sustain. Energy Rev..

[33] Motevali A., Tabatabaee Koloor R. (2017). A comparison between pollutants and greenhouse gas emissions from operation of different dryers based on energy consumption of power plants. J. Clean. Prod..

[34] Lohani Umesh C., Muthukumarappan K. (2017). Modeling of continuous ultrasonication to improve total phenolic content and antioxidant activity in sorghum flour: A comparison between response surface methodology and artificial neural network. Int. J. Food Eng..

[35] Nasirahmadi A., Emadi B., Abbaspour-Fard M.H., Aghagolzade H. (2014). Influence of moisture content, variety and parboiling on milling quality of rice grains. Rice Sci..

[36] Islam M.R., Shimizu N., Kimura T. (2004). Energy requirement in parboiling and its relationship to some important quality indicators. J. Food Eng..

[37] Paengkanya S., Soponronnarit S., Nathakaranakule A. (2015). Application of microwaves for drying of durian chips. Food Bioprod. Process..

[38] Kayran S., Doymaz İ. (2017). Determination of drying kinetics and physicochemical characterization of apricot pomace in hot-air dryer. J. Therm. Anal. Calorim..

[39] Aydogdu A., Sumnu G., Sahin S. (2015). Microwave—Infrared combination drying of eggplants. Food Bioprocess Technol..

[40] Khoshtaghaza M.H., Darvishi H., Minaei S. (2015). Effects of microwave—Fluidized bed drying on quality, energy consumption and drying kinetics of soybean kernels. J. Food Sci. Technol..

[41] Doymaz İ., Karasu S., Baslar M. (2016). Effects of infrared heating on drying kinetics, antioxidant activity, phenolic content, and color of jujube fruit. J. Food Meas. Charact..

[42] Jafari H., Kalantari D., Azadbakht M. (2018). Energy consumption and qualitative evaluation of a continuous band microwave dryer for rice paddy drying. Energy.

[43] Salarikia A., Miraei Ashtiani S.-H., Golzarian M.R. (2017). Comparison of drying characteristics and quality of peppermint leaves using different drying methods. J. Food Process. Preserv..

[44] Lechtańska J.M., Szadzińska J., Kowalski S.J. (2015). Microwave- and infrared-assisted convective drying of green pepper: Quality and energy considerations. Chem. Eng. Process. Process Intensif..

[45] Singh N., Paul P., Virdi A.S., Kaur P., Mahajan G. (2014). Influence of early and delayed transplantation of paddy on physicochemical, pasting, cooking, textural, and protein characteristics of milled rice. Cereal Chem..

[46] Tirawanichakul S., Bualuang O., Tirawanichakul Y. (2012). Study of drying kinetics and qualities of two parboiled rice varieties: Hot air convection and infrared irradiation. Songklanakarin J. Sci. Technol..

[47] Sareepuang K., Siriamornpun S., Wiset L., Meeso N. (2008). Effect of soaking temperature on physical, chemical and cooking properties of parboiled Fragrant rice. World J. Agric. Sci..

[48] Ayamdoo A.J., Demuyakor B., Dogbe W., Owusu R., Ofosu M.A. (2013). Effect of varying parboiling conditions on physical qualities of Jasmine 85 and Nerica 14 rice varieties. Am. J. Food Technol..

[49] Ahmadi Ghavidelan M., Amiri Chayjan R. (2016). Modeling engineering characteristics of hazelnut kernel during infrared fluidized bed drying. Food Meas..

[50] Elbert G., Tolaba M.P., Suárez C. (2001). Effects of drying conditions on head rice yield and browning index of parboiled rice. J. Food Eng..

[51] Lv B., Li B., Chen S., Chen J., Zhu B. (2009). Comparison of color techniques to measure the color of parboiled rice. J. Cereal Sci..

[52] Lamberts L., Brijs K.M.R., Verhelst N., Delcour J.A. (2006). Impact of browning reactions and bran pigments on color of parboiled rice. J. Agric. Food Chem..

[53] Parnsakhorn S., Noomhorm A. (2008). Changes in physicochemical properties of parboiled brown rice during heat treatment. Agric. Eng. Int. CIGR J..

