# Supplementary material for: Parboiled Paddy Drying with Different Dryers: Thermodynamic and Quality Properties, Mathematical Modeling Using ANNs Assessment
Source: Foods. 2020 Jan 13;9(1):86. doi: 10.3390/foods9010086 (PMC7023440; doi:10.3390/foods9010086)
Supplement: Supplementary file 1 [file foods-09-00086-s001.pdf]

Supplementary Materials

Figure S1: Schematic view of the experimental infrared convective-microwave drying: (1) fan and electric motor, (2) electrical heater, (3) duct and air tunnel, (4) infrared-convective chamber, (5) infrared lamp, (6) inverter and thermostat, (7) microwave chamber, (8) precision balance, (9) computer, (10) thermometer, (11) hygrometer, and (12) chassis

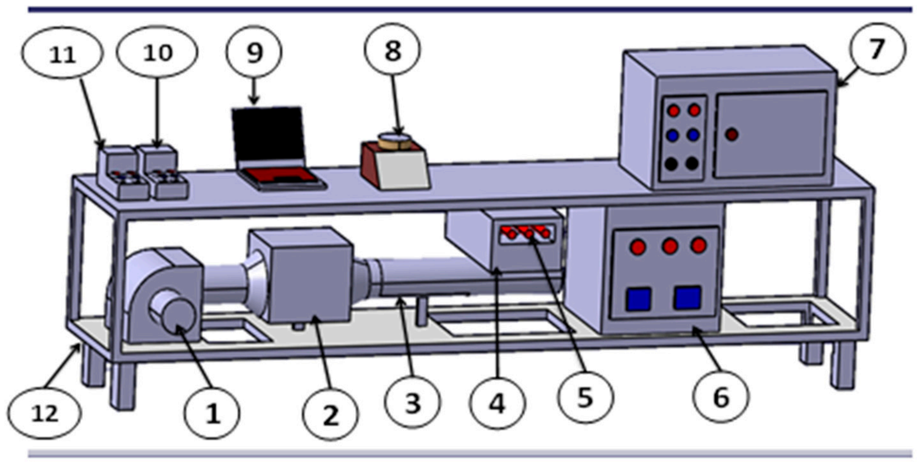

Figure S2: Effect of drying different methods on MR during drying of parboiled rice ((a): IRC drying; (b): IRCM drying; (c): MIC drying)

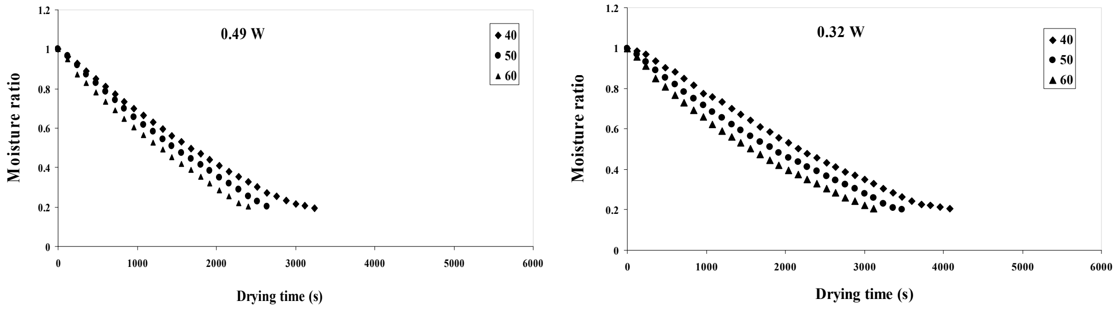

(a)

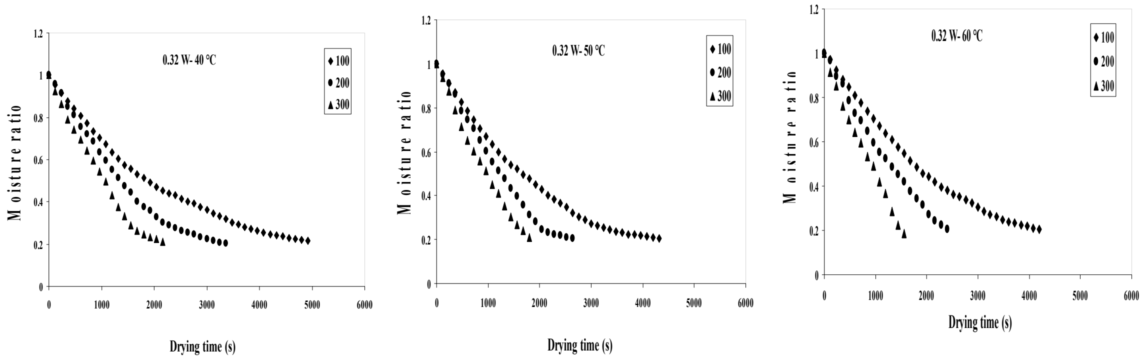

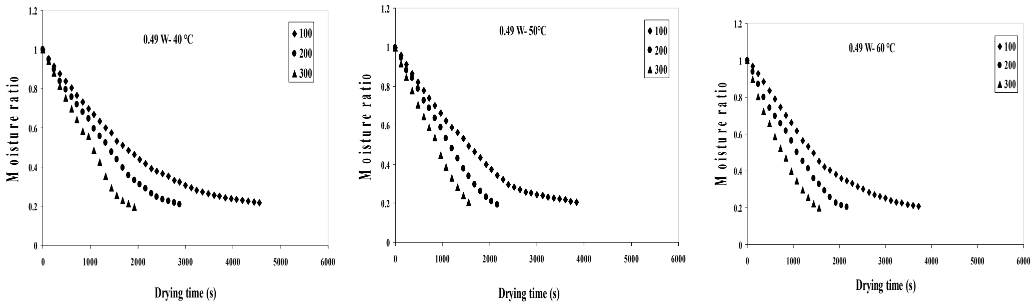

(b)

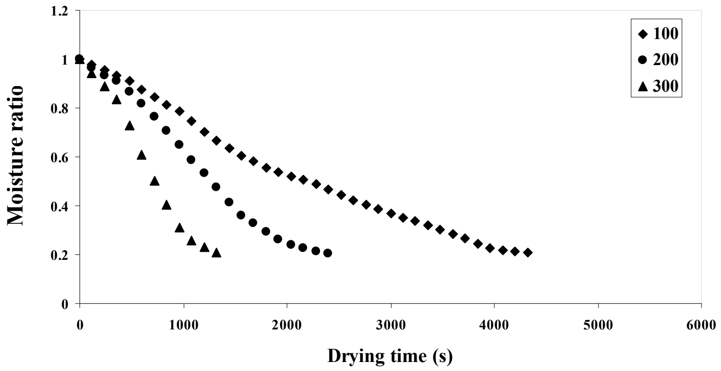

(C)

Table S1: Statistical comparison for prediction of MR of parboiled paddy samples under different drying conditions (RI= radiation intensity (w/ cm²), T= temperature (°C))

Model		R^2			χ^2			$RMSE$		
		**Infrared – convective drying**								
	**RI (w/ cm^2)**	**40 °C**	**50 °C**	**60 °C**	**40 °C**	**50 °C**	**60 °C**	**40 °C**	**50 °C**	**60 °C**
**Aghbashlo**	0.32	0.9999	0.9998	0.9998	0.0004	0.0012	0.0016	0.0194	0.0211	0.0221
	0.49	0.9996	0.9996	0.9994	0.0020	0.0022	0.0041	0.0229	0.0232	0.0274
Page	0.32	0.9993	0.9966	0.9960	0.0045	0.0267	0.0299	0.0282	0.0681	0.0741
	0.49	0.9969	0.9959	0.9974	0.0238	0.0311	0.0199	0.0655	0.0759	0.0584
Logestic	0.32	0.9994	0.9993	0.9988	0.0039	0.0059	0.0103	0.0268	0.0302	0.0398
	0.49	0.9989	0.9987	0.9981	0.0082	0.0112	0.0162	0.0362	0.0410	0.0513
Demir	0.32	0.9971	0.9944	0.9921	0.0222	0.0401	0.0511	0.0629	0.0886	0.0974
	0.49	0.9962	0.9952	0.9911	0.0288	0.0344	0.0585	0.0709	0.0812	0.1065

Midili	0.32	0.9997	0.9995	0.9994	0.0018	0.0028	0.0031	0.0224	0.0240	0.0251
	0.49	0.9994	0.9993	0.9989	0.0034	0.0053	0.0091	0.0257	0.0291	0.0380
		**Infrared – convective – microwave drying**								
	**RI (w/ cm^2) - T.(°C)**	**100 w**	**200 w**	**300 w**	**100 w**	**200 w**	**300 w**	**100 w**	**200 w**	**300 w**
**Aghbashlo**	0.32-40	0.9997	0.9999	0.9991	0.0012	0.0001	0.0008	0.0319	0.0102	0.0258
	0.32-50	0.9996	0.9995	0.9994	0.0013	0.0017	0.0017	0.0341	0.0380	0.0380
	0.32-60	0.9970	0.9984	0.9982	0.0029	0.0010	0.0009	0.0491	0.0290	0.0276
	0.49-40	0.9994	0.9994	0.9994	0.0039	0.0035	0.0032	0.0562	0.0529	0.0513
	0.49-50	0.9995	0.9994	0.9996	0.0026	0.0028	0.0020	0.0455	0.0473	0.0396
	0.49-60	0.9997	0.9997	0.9996	0.0012	0.0010	0.0012	0.0315	0.0288	0.0306
Page	0.32-40	0.9962	0.9962	0.9962	0.0257	0.0254	0.0244	0.1526	0.1511	0.1481
	0.32-50	0.9961	0.9952	0.9948	0.0211	0.0251	0.0253	0.1374	0.1493	0.1499
	0.32-60	0.9926	0.9918	0.9944	0.0111	0.0152	0.0043	0.1008	0.1177	0.0623
	0.49-40	0.9953	0.9938	0.9932	0.0175	0.0224	0.0235	0.1270	0.1434	0.1468
	0.49-50	0.9943	0.9933	0.9928	0.0117	0.0132	0.0136	0.1037	0.1101	0.1114
	0.49-60	0.9959	0.9962	0.9954	0.0175	0.0157	0.0182	0.1247	0.1178	0.1268
Logestic	0.32-40	0.9983	0.9980	0.9979	0.0125	0.0128	0.0125	0.1035	0.1043	0.1034
	0.32-50	0.9982	0.9976	0.9974	0.0092	0.01207	0.0127	0.0883	0.1002	0.1031
	0.32-60	0.9979	0.9978	0.9977	0.0095	0.0084	0.0096	0.0892	0.0834	0.0890
	0.49-40	0.9978	0.9967	0.9964	0.0086	0.0112	0.0117	0.0871	0.0990	0.1015
	0.49-50	0.9980	0.9976	0.9974	0.0029	0.0034	0.0028	0.0505	0.0545	0.0499
	0.49-60	0.9968	0.9985	0.9977	0.0048	0.0009	0.0017	0.0645	0.0291	0.0383
Demir	0.32-40	0.9990	0.9989	0.9991	0.0034	0.0034	0.0031	0.0544	0.0548	0.0522
	0.32-50	0.9984	0.9985	0.9989	0.0084	0.0074	0.0051	0.0833	0.0778	0.0649
	0.32-60	0.9986	0.9982	0.9983	0.0037	0.0071	0.0067	0.0554	0.0758	0.0733

	0.49-40	0.9983	0.9984	0.9985	0.0112	0.0101	0.0094	0.0973	0.0921	0.0881
	0.49-50	0.9988	0.9976	0.9987	0.0016	0.0056	0.0014	0.0380	0.0694	0.0349
	0.49-60	0.9976	0.9988	0.9983	0.0035	0.0008	0.0012	0.0546	0.0272	0.0327
Midili	0.32-40	0.9989	0.9998	0.9816	0.0015	0.0002	0.0208	0.0361	0.0140	0.1305
	0.32-50	0.9957	0.9938	0.9931	0.0160	0.0223	0.0238	0.1161	0.1363	0.1408
	0.32-60	0.9977	0.9812	0.9839	0.0033	0.0053	0.0125	0.0524	0.0664	0.1005
	0.49-40	0.9986	0.9987	0.9988	0.0084	0.0082	0.0075	0.0829	0.0811	0.0775
	0.49-50	0.9761	0.9729	0.9694	0.0717	0.1126	0.1225	0.2361	0.2934	0.3059
	0.49-60	0.9791	0.9769	0.9771	0.1115	0.1196	0.1152	0.2966	0.3049	0.2993
			**Microwave drying**							
		**100 w**	**200 w**	**300 w**	**100 w**	**200 w**	**300 w**	**100 w**	**200 w**	**300 w**
**Aghbashlo**		0.9999	0.9994	0.9993	0.0006	0.0021	0.0027	0.0210	0.0249	0.0251
Page		0.9986	0.9964	0.9948	0.0077	0.0194	0.0272	0.0304	0.0501	0.0596
Logestic		0.9989	0.9985	0.9970	0.0057	0.0084	0.0148	0.0279	0.0313	0.0410
Demir		0.9982	0.9953	0.9922	0.0116	0.0243	0.0324	0.0359	0.0564	0.0618
Midili		0.9995	0.9990	0.9978	0.0018	0.0051	0.0127	0.0231	0.0268	0.0388
